# Lipid Abnormalities in Patients With Cushing’s Disease and Its Relationship With Impaired Glucose Metabolism

**DOI:** 10.3389/fendo.2020.600323

**Published:** 2021-02-09

**Authors:** Xiaolin Sun, Ming Feng, Lin Lu, Zixuan Zhao, Xinjie Bao, Kan Deng, Yong Yao, Huijuan Zhu, Renzhi Wang

**Affiliations:** ^1^ Department of Neurosurgery, Chinese Academy of Medical Sciences and Peking Union Medical College, Peking Union Medical College Hospital, Beijing, China; ^2^ School of Medicine, Tsinghua University, Beijing, China; ^3^ Department of Endocrinology, Chinese Academy of Medical Sciences and Peking Union Medical College, Peking Union Medical College Hospital, Beijing, China

**Keywords:** Cushing’s disease, hyperlipidemia, lipid profile, glucose metabolism, hypercortisolism

## Abstract

**Purpose:**

Dyslipidemia has been frequently reported and associated with increased cardiovascular risk in patients with Cushing’s disease (CD). Few studies are available regarding the relationships between lipid abnormalities and other preoperative metabolic comorbidities in CD, and the data on alterations of the lipid profile after surgery is quite variable. We aimed to investigate the associations between hyperlipidemia and other baseline metabolic and hormonal parameters and the impact of surgical remission on lipid metabolism in patients with CD.

**Methods:**

This retrospective study included 104 patients diagnosed with CD. Baseline hormonal and metabolic parameters were compared between the hyperlipidemia (HLP) group and non-hyperlipidemia (NLP) group, and their relationships with hyperlipidemia at diagnosis were evaluated. Alterations in lipid profiles after surgical remission of CD were evaluated in 65 patients with available follow-up data.

**Results:**

Upon baseline, logistic regression analysis showed that impaired glucose metabolism (IGM) (OR=4.68, 95%CI:1.38–15.91) and morning cortisol levels (per 10 μg/dl change) (OR=1.81, 95%CI:1.11–2.95) are both independent risk factors of preoperative occurrence of hyperlipidemia in patients with CD. The baseline triglyceride (TG) level was positively correlated with systolic blood pressure (SBP) (r=0.297, p=0.003). Lipid abnormalities had improvement but may persist after surgical remission, and the persisted hyperlipidemia is associated with higher baseline total cholesterol (TC) levels (r=0.505, p=0.033).

**Conclusions:**

Persistence of post-surgery hyperlipidemia is associated with severe baseline lipid abnormalities. Surgical remission with concomitant control of impaired glucose metabolism at diagnosis may have significant implications for controlling hyperlipidemia and reducing cardiovascular risk in CD.

## Introduction

Cushing’s disease (CD) is the most prevalent etiology of Cushing’s syndrome (CS), presenting with endogenous hypercortisolism caused by an ACTH-secreting pituitary tumor (1). It is characterized by a series of metabolic disorders, including visceral obesity, hypertension, impaired glucose metabolism, and dyslipidemia, leading to increased cardiovascular risk and a higher mortality rate compared with the healthy population (2, 3).

Dyslipidemia secondary to chronic glucocorticoid excess has been reported in 12%–72% of CS patients and was a critical contributor to the increased cardiovascular complications, such as vascular atherosclerosis, coronary artery disease, and heart failure (1). The lipid abnormalities associated with CS usually present as elevated total cholesterol (TC), low-density lipoprotein cholesterol (LDL-c) and triglyceride (TG) levels, and lower high-density lipoprotein cholesterol (HDL-c) level in patients compared with healthy controls and may persist despite the surgical remission of CS (1, 4, 5). However, the alterations of the lipid profile after surgical correction of hypercortisolism is quite variable in previous studies (1, 4).

Furthermore, the pathogenic mechanisms of lipid abnormalities in CS, particularly in CD, are complex and remained largely unknown. Previous studies have shown that glucocorticoids (GC) excess could stimulate both lipolysis and lipogenesis, resulting in increased intravascular triglyceride hydrolysis, hepatic free fatty acid (FFA) production, and very-low-density lipoprotein cholesterol (VLDL-c) synthesis whereas suppression of FFA oxidation (5–7). Hyperglycemia and insulin resistance, as common features in CD, were suggested to play critical roles in determining lipid dysregulation in non-CD populations (e.g., type 2 diabetes mellitus) (5, 8). However, few studies are available regarding the relationships between lipid abnormalities and other preoperative metabolic comorbidities in CD patients (9).

Therefore, this study aimed to evaluate the potential associations between metabolic and hormonal parameters and hyperlipidemia associated with CD at the time of diagnosis. Besides, we investigated the short-term impact of surgical remission on lipid metabolism and assessed the potential parameters that predict the persistence of hyperlipidemia after remission of CD at follow-up.

## Methods

### Patients

This retrospective study included 104 patients diagnosed with Cushing’s disease (CD) and hospitalized at Peking Union Medical College Hospital, Chinese Academy of Medical Science, from April 2013 to September 2019. Patients were included in the study if newly diagnosis of CD was made based on: typical Cushingoid appearance, e.g., moon face, acne, supraclavicular, and dorsal fat pads; elevated morning serum or 24-h urinary free cortisol (24hUFC) level, absence of circadian cortisol rhythm, increased plasma ACTH level; lack of suppression of low-dose dexamethasone suppression test (LDDST) whereas suppressibility with high dexamethasone suppression test (HDDST); detection of a pituitary lesion on MRI. All patients underwent endoscopic endonasal transsphenoidal surgery for pituitary adenoma resection. The adenoma was carefully separated and completely resected for each patient, and then followed by nasoseptal flap reconstruction of the diaphragm and sellar. The diagnosis was confirmed by postoperative pathology and immunohistological staining results, indicating a pituitary adenoma with ACTH (+).

**Figure 1:**
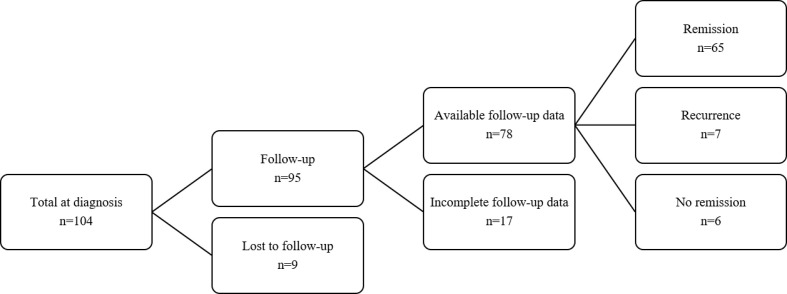
Follow-up flow chart of CD patients included in the study.

Patients were excluded if: 1) recurrent CD with previous surgery or radiotherapy; 2) complete medical data at baseline was not available; 3) administration of drugs influencing cortisol metabolism or usage of hypolipidemic medication at the time of diagnosis. Data on clinical and biochemical parameters at diagnosis of CD and at the last follow-up visit were retrospectively extracted from the medical records. A total of 104 patients was included in the investigation of baseline characteristics (**Figure 1**). Of these, 95 patients were followed up for at least 6 months after surgery, while nine patients were lost to follow-up. Re-evaluation of lipid profile at last follow-up was available in 78 patients, of whom 65 achieved remission and 6 had no remission that constituted the cohort for comparison analysis between baseline and post-surgery parameters.

Disease duration was calculated as the period from symptom onset to biochemical remission of hypercortisolism. Immediate surgical remission was defined as a serum cortisol level <5 µg/dl measured within the postoperative 7 days (10, 11). Low-dose glucocorticoid was routinely given to patients with immediate surgical remission and gradually ceased within 3 months to help reducing adverse effects of temporary hypopituitarism. Long-term hydrocortisone replacement therapy (HRT) was required for patients with postoperative hypopituitarism or adrenal insufficiency. Female patients with postoperative hypogonadotropic hypogonadism (HH) were given low-dose estradiol additionally. At follow-up, remission was considered when patients had normalized serum cortisol and 24hUFC levels along with the resolution of clinical symptoms. Recurrence was defined as elevated serum cortisol or 24hUFC above the upper limit of normal range companied by clinical symptoms in patients with initially established remission.

Informed consent was obtained from each patient. This study was approved by the Institutional Review Board of Peking Union Medical College Hospital, Chinese Academy of Medical Sciences and was performed following the ethical standards laid down in the 1964 Declaration of Helsinki and its later amendments.

### Clinical and Biochemical Evaluation

Body mass index (BMI) was calculated using the formula weight (kg)/height^2^ (m^2^). Obesity was defined using BMI criteria for Asians as BMI≥28 kg/m^2^. Waist circumference above 88 cm in women and 102 cm in men was defined as visceral obesity. Blood pressure was measured in all patients twice a day, and the average of two measurements was calculated. Hypertension was defined as systolic blood pressure (SBP) ≥140 mmHg or diastolic blood pressure (DBP)≥90 mmHg, or current use of antihypertensive medication. Impaired glucose metabolism (IGM) was defined when impaired glucose tolerance(IGT) (2h glucose after OGTT between 7.8 and 11.0 mmol/L) or diabetes mellitus (DM) (fasting blood glucose ≥7.0 mmol/L or 2h glucose after OGTT ≥11.1 mmol/L) was diagnosed according to ADA 2014 guidelines, or when patients were on current antidiabetic medication. Hypercholesterolemia was diagnosed when total cholesterol (TC) ≥6.22 mmol/L, whereas hypertriglyceridemia was diagnosed when triglyceride (TG) ≥2.26 mmol/L according to the NCEP-ATPIII criteria. The presence of either hypercholesterolemia or hypertriglyceridemia was summarized as hyperlipidemia. All biochemical parameters were evaluated by standard procedures in the routine certified laboratory of our institution. TC, TG, HDL-cholesterol, LDL-cholesterol were determined using Beckman AU5400 automatic biochemistry analyzer and homogeneous methods. Serum cortisol, serum ACTH, 24hUFC, and other hormones were measured with the chemiluminescence method using the commercial kits (DPC Biotechnology and Medical Products Cooperation, Tianjin, China). Glycated hemoglobin (HbA1c) was measured with high performance liquid chromatography. According to the WHO diagnostic guidelines, osteoporosis was defined as low bone density as measured using dual-energy X-ray absorptiometry (DEXA). Hepatic steatosis and atherosclerosis were examined using high-resolution B-mode ultrasonography. Cardiovascular disease (CVD) history was defined as a medical history of myocardial infarction, unstable angina, ischemic stroke, cardiac structural changes, or severe arrhythmia. Patients reported a family history of hypertension or DM in first-degree relatives was considered to have hypertension or DM family history. Patients with long-term HRT were asked to be off their hormonal medication for 48h before blood collection at follow-up.

### Statistical Analysis

The normality of data distribution was determined using the Shapiro-Wilks test. Quantitative variables were presented as mean ± standard deviations(SD) or median with interquartile range(IQRs) according to their distribution, and categorical variables were reported as frequency. Differences among independent groups were analyzed using Student’s t-tests for quantitative data with normal distribution, or Mann-Whitney U tests for data with non-normal distribution. Pearson’s Chi-square test was used to compare the differences for categorical variables. Paired t-test or Wilcoxon signed-rank test was used to compare the differences between pre- and post-operation parameters for quantitative variables, whereas the McNemar test was used for categorical variables. Spearman’s correlation coefficients and Partial correlation coefficients were used for correlation analyses. Multivariate logistic regression analysis was used to investigate associations of baseline parameters with the presence of hyperlipidemia before surgery. Data analysis was performed with Statistical Package for Social Sciences (SPSS) Version 25.0 program (IBM Corporation, Armonk, NY, USA). All statistical tests were two-sided, and p < 0.05 was considered statistically significant.

## Results

Upon diagnosis, hyperlipidemia, hypertension, and impaired glucose metabolism (IGM) were present in 36.4%, 73.1%, and 70.2% of patients with CD, respectively. One hundred and four patients with CD were categorized into two groups: non-hyperlipidemia (NLP) group (n=68, 65.4%) and hyperlipidemia (HLP) group (n=36, 34.6%). The baseline characteristics of the two groups are shown in **Table 1**. Patients with HLP showed higher systolic blood pressure (SBP), 2h-blood glucose during OGTT (2h-BG), morning serum cortisol levels compared with NLP patients (p<0.05). Also, the prevalence of IGM (88.9% vs. 60.3%, p=0.002) and diabetes mellitus (DM) family history (19.4% vs. 4.4%, p=0.03) tended to be higher in patients with HLP than that in NLP patients. There were no significant differences in age, body mass index (BMI), disease duration, diastolic blood pressure (DBP), high-density lipoprotein cholesterol (HDL-c), tumor size, plasma ACTH between the two groups. Other pre-operative comorbidities, including visceral obesity, osteoporosis, hepatic steatosis, and atherosclerosis, were also comparable between groups regardless of the HLP status. An association was detected between baseline triglycerides (TG) and SBP (r=0.297, p=0.003), and fasting blood glucose (FBG) (r=0.338, p=0.001), and HbA1c (r=0.299, p=0.004), respectively, after adjustment for all covariates including age, BMI, serum cortisol level, plasma ACTH level, and disease duration. Furthermore, the logistic regression analysis showed that IGM (OR=4.682, 95%CI: 1.378–15.909) and morning serum cortisol level (per 10 μg/dl change) (OR=1.807, 95%CI: 1.107–2.949) are independent risk factors of the preoperative presence of hyperlipidemia as a comorbidity in patients with CD regardless of age, obesity and disease duration (**Table 2**).

**Table 1:** Baseline characteristics at diagnosis in CD patients with or without hyperlipidemia.

	Total	NLP	HLP	p
Patient(n/%)	104(100%)	68(65.4%)	36(34.6%)	–
Age(years)	33.02 ± 11.73	32.41 ± 10.51	34.17 ± 13.81	0.508
Gender(Female)(n/%)	85(81.7%)	53(77.9%)	32(88.9%)	0.194
BMI(kg/m2)	26.55(25.07–28.86)	26.28(24.97–28.44)	27.14(25.74–30.18)	0.158
Obesity(n/%)	34(32.7%)	19(27.9%)	15(41.7%)	0.156
Disease duration (months)	24.00(12.00–60.00)	24.00(12.75–60.00)	24.00(10.50–81.00)	0.683
SBP(mmHg)	139.35 ± 17.69	136.65 ± 16.51	144.44 ± 18.93	0.032*
DBP(mmHg)	92.40 ± 14.65	91.85 ± 13.98	93.44 ± 16.01	0.601
HT(n/%)	76(73.1%)	48(70.6%)	28(77.8%)	0.432
TC(mmol/L)	5.74 ± 1.15	5.13 ± 0.72	6.91 ± 0.85	<0.001*
TG(mmol/L)	1.43(0.98–2.00)	1.24(0.89–1.65)	2.24(1.54–3.34)	<0.001*
HDL-c(mmol/L)	1.40(1.19–1.64)	1.40(1.19–1.62)	1.41(1.16–1.74)	0.534
LDL-c(mmol/L)	3.53(3.01–4.04)	3.21(2.58–3.58)	4.14(3.81–4.73)	<0.001*
Total/HDL-c Ratio	3.97(3.28–4.98)	3.77(3.17–4.34)	5.01(3.85–5.61)	<0.001*
FBG(mmol/L)	4.90(4.50–5.63)	4.80(4.48–5.40)	5.20(4.50–6.08)	0.102
2h-BG(mmol/L)	9.45(7.18–13.68)	8.85(6.60–13.68)	10.45(8.65–14.03)	0.015*
HbA1c(%)	5.50(5.20–5.90)	5.50(5.10–5.90)	5.70(5.30–6.30)	0.108
IGM(n/%)	73(70.2%)	41(60.3%)	32(88.9%)	0.002*
F[8am](μg/dl)	27.30(20.70–32.36)	24.98(19.97–31.36)	29.98(22.81–36.93)	0.041*
ACTH(pg/ml)	63.60(49.45–97.55)	59.45(43.85–95.43)	66.85(54.23–107.75)	0.241
24hUFC(μg)	412.5(264.17–614.28)	417.55(265.34–630.3)	367.2(254.75–557.82)	0.564
Tumor size(mm)	5.40(4.63–7.98)	5.40(4.70–7.65)	5.25(4.45–8.75)	0.986
Visceral obesity(n/%)	79(76.0%)	48(70.6%)	31(86.1%)	0.095
Osteoporosis(n/%)	52(50%)	36(52.9%)	16(44.4%)	0.410
Hepatic steatosis(n/%)	33(31.7%)	18(26.5%)	15(41.7%)	0.113
Atherosclerosis(n/%)	11(10.6%)	7(10.3%)	4(11.1%)	1.000
CVD history(n/%)	13(12.5%)	6(8.8%)	7(19.4%)	0.119
HT family history(n/%)	32(30.8%)	17(25.0%)	15(41.7%)	0.080
DM family history(n/%)	10(9.6%)	3(4.4%)	7(19.4%)	0.030*
Antihypertensive medication(n/%)	58(55.8%)	35(51.5%)	23(63.9%)	0.225
Antidiabetic medication (n/%)	12(11.5%)	9(13.2%)	3(8.3%)	0.537

HLP, hyperlipidemia; NLP, non-hyperlipidemia; BMI, body mass index; SBP, systolic blood pressure; DBP, diastolic blood pressure; HT, hypertension; TC, total cholesterol; TG, triglycerides; HDL-c, high-density lipoprotein cholesterol; LDL-c, low-density lipoprotein cholesterol; FBG, fasting blood glucose; 2h-BG, 2h-blood glucose during OGTT; IGM, impaired glucose metabolism; F_[8am]_, serum cortisol (8am); ACTH, adrenocorticotropic hormone; 24hUFC, 24-h urine free cortisol; CVD, cardiovascular disease; DM, diabetes mellitus. *Statistically significant.

**Table 2:** Logistic regression analyses of hyperlipidemia at diagnosis with predicting baseline parameters.

	OR	95% CI	p
Age≥38y	1.249	0.480–3.254	0.649
Obesity	1.676	0.646–4.346	0.288
IGM	4.682	1.378–15.909	0.013*
F_[8am]_ (10 µg/dl change)	1.807	1.107–2.949	0.018*
Disease duration(months)	1.007	0.996–1.017	0.206

OR, odd ratios; CI confidence interval; IGM, impaired glucose metabolism; F_[8am]_, serum cortisol (8am). *Statistically significant.

After surgery, immediate remission in postoperative 7 days was achieved in 85.6% (89/104) patients. No significant difference in prevalence of surgical complications (16.2% vs. 19.4%, p=0.892) and immediate postoperative remission (85.2% vs. 86.1%, p=1.000) was found between NLP and HLP groups. At the last visit, a total of 78 patients had available biochemical data, of which 65 achieved remission after surgery. Improvement in lipid profile with a significant reduction in total cholesterol (TC) and low-density lipoprotein cholesterol (LDL-c) level was observed in these 65 patients at follow-up when compared with baseline levels (p<0.001) (**Table 3**). Moreover, a tendency towards lower TG level after surgical remission was showed in comparison with baseline, although not statistically significant. Surprisingly, the level of HDL-c also showed a decrease at follow-up in remission group. The prevalence of hyperlipidemia (33.8% vs. 20.0%, p=0.049) and hypercholesterolemia was significantly decreased at follow-up (32.3% vs. 10.8%, p=0.001) whereas that of hypertriglyceridemia remained unchanged. Additionally, the level of HbA1c at follow-up was lower than that of pre-surgery. However, there was no significant change in lipid profile in six non-remission patients after surgery. As for the change rates of lipid profile and dyslipidemia status before and after surgery, the prevalence of hyperlipidemia and hypercholesterolemia was observed significantly decreased in remission group compared with the non-remission group (**Table 3**). Out of 36 patients with preoperative hyperlipidemia, 22 patients had biochemical remission after surgery with available data on lipid profile at last follow-up, which was further classified into 2 groups according to their change of hyperlipidemia status: the improved group (n=13, from HLP to NLP), and the persisted group (n=9, from HLP to HLP). When comparing the baseline characteristics between the two groups (**Table 4**), the persisted group was presented with higher baseline TC than the improved group(p=0.033). Baseline TG and LDL-c levels were also tended to be higher in the persisted group than in the improved group, although the difference did not reach statistical significance. However, there were no differences concerning age, BMI, disease duration, IGM, serum cortisol, plasma ACTH, the percentage of hypertension, post-surgery growth hormone deficiency (GHD), post-surgery hypogonadotropic hypogonadism (HH), and hydrocortisone replacement therapy (HRT) between the two groups. Correlation analysis showed that the higher baseline TC level was significantly associated with the persistence of hyperlipidemia after surgery (r=0.505, p=0.033) independent of age, BMI, disease duration, and preoperative serum cortisol level.

**Table 3:** Baseline and post-surgery changes in CD patients with remission or no remission at follow-up.

	Remission (n=65)		Non-Remission (n=6)		p^a^	p^b^	p^c^
	Baseline	Post-surgery	Baseline	Post-surgery			
Surgical complications(n/%)	–	17(26.2%)	–	0(0.0%)	–	–	0.324
GHD	–	9(13.8%)	–	0(0.0%)	–	–	1.000
HH	–	8(12.3%)	–	0(0.0%)	–	–	1.000
DI	–	1(5.9%)	–	0(0.0%)	–	–	1.000
Follow-up(months)	–	16.83(12.7–23.40)	–	15.11(8.09–17.88)	–	–	0.217
F_[8am]_(μg/dl)	27.21(20.55–33.95)	8.90(5.30–11.55)	21.75(16.43–29.04)	30.3(28.4–33.19)	<0.001*	0.063	<0.001*
ACTH(pg/ml)	61.70(45.25–97.00)	16.70(9.30–25.15)	52.25(39.35–56.80)	43.55(26.58–61.08)	<0.001*	0.438	<0.001*
24hUFC(μg)	433.30(285.28–609.35)	29.55(16.70–41.98)	298.11(196.95–483.20)	280.15(162.15–649.49)	<0.001*	1.000	<0.001*
TC(mmol/L)	5.70 ± 1.27	4.83 ± 1.12	5.63 ± 0.79	5.68 ± 1.15	<0.001*	0.844	0.121
TG(mmol/L)	1.42(0.96–1.88)	1.16(0.86–1.85)	1.05(0.93–1.42)	1.09(0.90–1.27)	0.153	1.000	0.678
HDL-c(mmol/L)	1.42(1.20–1.71)	1.14(0.99–1.35)	1.33(1.11–1.40)	1.54(1.13–1.62)	<0.001*	0.438	0.023*
LDL-c(mmol/L)	3.37(2.71–4.11)	2.92(2.32–3.45)	3.65(3.24–4.26)	3.93(2.94–4.55)	<0.001*	1.000	0.276
Total/HDL-c Ratio	3.95(3.17–4.80)	4.03(3.31–5.03)	4.34(3.92–5.40)	4.00(3.34–4.64)	0.339	0.125	0.253
Hyperlipidemia(n/%)	22(33.8%)	13(20.0%)	1(16.7%)	3(50.0%)	0.049*	0.625	0.012*
Hypercholesterolemia(n/%)	21(32.3%)	7(10.8%)	1(16.7%)	3(50.0%)	0.001*	0.625	0.004*
Hypertriglyceridemia(n/%)	8(12.3%)	9(13.8%)	0(0.0%)	0(0.0%)	1.000	1.000	1.000
HbA1c(%)	5.50(5.20–5.90)	5.30(4.93–5.50)	5.50(5.25–5.80)	5.40(5.33–5.55)	0.001*	0.625	0.935

GHD, growth hormone deficiency; HH, Hypogonadotropic hypogonadism; DI, diabetes insipidus; F_[8am]_, serum cortisol (8am); ACTH, adrenocorticotropic hormone; TC, total cholesterol; TG, triglycerides; HDL-c, high-density lipoprotein cholesterol; LDL-c, low-density lipoprotein cholesterol. *Statistically significant. p^a^, p value for comparing baseline and post-surgery characteristics in remission group. p^b^, p value for comparing baseline and post-surgery characteristics in non-remission group. p^c^, p value for comparing the change rates of each parameters before and after surgery between remission and non-remission group.

**Table 4:** Comparison of baseline and post-surgery characteristics of CD patients with biochemical remission after surgery presenting with improved or persisted hyperlipidemia status at last follow-up.

	Change in hyperlipidemia status		
	Improved	Persisted	p
Patient(n/%)	13(51.9%)	9(48.1%)	–
Follow-up(months)	19.55(16.89–26.85)	19.10(14.54–20.00)	1.000
Age(years)	32.54 ± 15.92	36.56 ± 11.78	0.246
Gender(Female)(n/%)	12(92.3%)	8(88.9%)	0.784
BMI(kg/m^2^)	27.39(24.72–29.31)	26.39(23.39–28.55)	0.647
Disease duration (months)	34.00(7.00–102.50)	24.00(9.00–36.00)	0.471
SBP(mmHg)	139.46 ± 19.51	146.56 ± 12.30	0.645
DBP(mmHg)	91.38 ± 16.16	93.56 ± 12.05	0.766
HT(n/%)	7(53.8%)	8(88.9%)	0.165
TC(mmol/L)	6.70 ± 0.68	7.53 ± 1.04	0.033*
TG(mmol/L)	1.79(1.38–2.52)	2.19(1.40–3.16)	0.431
HDL(mmol/L)	1.44(1.24–1.73)	1.68(1.39–2.12)	0.393
LDL(mmol/L)	4.14(3.82–4.83)	4.42(4.09–5.17)	0.262
Total/HDL-c Ratio	4.98(3.66–5.09)	4.67(3.44–5.32)	0.948
FBG(mmol/L)	4.80(4.20–5.90)	4.90(4.65–5.85)	0.556
2h-BG(mmol/L)	10.20(8.45–11.25)	10.60(9.35–12.20)	0.431
HbA1c(%)	5.65(4.90–5.95)	5.80(5.35–6.15)	0.382
IGM(n/%)	12(92.3%)	9(100.0%)	1.000
F_[8am]_(μg/dl)	30.85(25.78–36.15)	21.30(19.18–32.60)	0.110
ACTH(pg/ml)	70.20(53.00–101.80)	56.20(54.20–78.10)	0.357
24hUFC(μg)	496.60(331.25–708.02)	357.28(202.30–443.73)	0.239
Tumor size(mm)	5.40(5.00–7.75)	5.50(3.95–8.45)	0.905
Visceral obesity(n/%)	12(92.3%)	6(66.7%)	0.264
HRT(n/%)	4(30.8%)	1(11.1%)	0.360
HRT Duration(months)	9.64(4.69–30.3)	30.28	0.800
Post-surgery GHD(n/%)	3(23.1%)	0(0.0%)	0.240
Post-surgery HH(n/%)	1(7.7%)	1(11.1%)	1.000

BMI, body mass index; SBP, systolic blood pressure; DBP, diastolic blood pressure; HT, hypertension; TC, total cholesterol; TG, triglycerides; HDL-c, high-density lipoprotein cholesterol; LDL-c, low-density lipoprotein cholesterol; FBG, fasting blood glucose; 2h-BG, 2h-blood glucose during OGTT; IGM, impaired glucose metabolism; F_[8am]_, serum cortisol (8am); ACTH, adrenocorticotropic hormone; 24hUFC, 24-h urine free cortisol; HRT, hydrocortisone replacement therapy. GHD, growth hormone deficiency; HH, Hypogonadotropic hypogonadism. *Statistically significant.

## Discussion

The present study identified both preoperative morning serum cortisol level and impaired glucose metabolism as independent risk factors of hyperlipidemia occurrence during the active phase of CD, suggesting that IGM and the degree of hypercortisolism may together contribute to the pathogenesis of hyperlipidemia in CD.

The mechanisms of hyperlipidemia in CD are multifactorial. It is well known that glucocorticoids (GC) have complex effects on lipid metabolism, including direct and indirect GC action on lipolysis and lipogenesis, adipocytes differentiation and distribution, free fatty acid (FFA) production and turnover, VLDL synthesis and hepatic fatty accumulation (4, 5, 12). Specifically, GC increases both intra-adipocyte and intravascular lipolysis by activating lipoprotein lipase, leading to increased triglyceride hydrolysis in adipose tissues and turnover of FFA in the circulation (5, 13, 14). On the other hand, GC enhances hepatic lipogenesis and VLDL production by stimulating lipogenic enzyme activities and inhibits FFA oxidation in the liver (5). In a previous study, patients with CS had increased fatty acid synthase expression than controls owing to the suppression of AMP-activated protein kinase (AMPK) activity in visceral adipose tissue, which of interest was correlated with the severity of hypercortisolism (15). Glucocorticoid inhibition of hairy enhancer of split 1 (Hes1) gene expression that regulates hepatic production of pancreatic lipase is also involved in the pathogenesis of dyslipidemia (16). Besides, the enzyme 11β-hydroxysteroid dehydrogenase type 1 (11βHSD-1) that regulates intracellular active GC concentrations was found increased in the adipose tissue of obese humans. Transgenic mice models expressing increased 11βHSD-1 activity selectively in either adipose tissue or liver would develop visceral obesity or hepatic steatosis respectively, and both exhibited visceral obesity and the metabolic syndrome, such as insulin-resistance, hyperlipidemia and hypertension (17, 18), which suggests that GC mediated by11βHSD-1 activity may play a significant role in adipose tissue redistribution and metabolic syndrome. Moreover, visceral obesity due to hypercortisolism in CD also plays an essential role in the determination of lipid abnormalities by promoting increased lipolysis, aberrant adipokine secretion, low-grade inflammation, and insulin resistance (19, 20). Previous data regarding the relationship between lipid profile and the degree of hypercortisolemia is very variable. Higher serum or urine cortisol level has been shown associated with high TG, high LDL-c, and low HDL-c levels (6, 21, 22), while Mancini et al. showed a reverse correlation between TC and LDL-c with morning serum cortisol levels in CS (23). Nevertheless, no correlation was found between serum morning cortisol and any lipid parameters in Dhingra’s study (24). In the current study, we found a significant positive correlation between TC and morning serum cortisol level, but no significant associations between the extent of cortisol excess and any other lipid parameters. Genetic variations in the glucocorticoid receptors (GR) can affect the function of GC and lipid metabolism. In particular, N363S polymorphism related to hypersensitivity to GC was recently associated with increased TC and TG levels and total/HDL-c ratio (6). The widely existed genetic polymorphism of GR may partially explain the inconsistency in associations between cortisol level and dyslipidemia in CD. The difference might also be due to various criteria for diagnosing dyslipidemia in different studies.

Additionally, insulin resistance is a primary underlying disorder that drives lipid abnormalities. It was revealed that in CD patients the serine kinases phosphorylating serine sites on insulin receptors, such as JNK and IKK-β, are activated, which results in insulin resistance (25), and in return, insulin resistance acts synergistically with hypercortisolism to upregulate lipogenesis, causing central obesity and hepatic steatosis (26).The main abnormality related to IGM is increased assembly and secretion of VLDL, apoB, and TG (27, 28). The Framingham Offspring Study demonstrated an association between diabetes and a higher prevalence of elevated TG and decreased HDL cholesterol (29). Inadequate glycemic control may exacerbate HDL dysfunction in patients with T2DM (30, 31). The current data showed that hyperlipidemia was significantly associated with the 2h-BG level as well as the prevalence of diabetes DM family history in CD. Insulin resistance affects all of the TG supply pathways for VLDL assembly, resulting in increased assembly and secretion of VLDL and the consequent hypertriglyceridemia, leading to reduced HDL-cholesterol levels and increased small dense LDL (sdLDL) particles (32, 33).

Although GC replacement therapy has been associated with increased TG, TC, and LDL concentrations in patients with hypopituitarism (34), a recent study by Choi et al. showed exogenous GC exposure has no association with lipid abnormalities (35). Of interest, in the absence of IGM, subclinical hypercortisolism does not affect lipid metabolism in patients with adrenal incidentalomas (36). The association between dyslipidemia with IGM rather than subtle GC excess suggests that GC may indirectly affect lipid metabolism by mediating glucose metabolism and insulin resistance.

In CD, hypercortisolism induces impairment of glucose metabolism by both direct and indirect effects, including stimulation of hepatic gluconeogenesis, inducement of insulin resistance by impairing insulin signaling pathway in peripheral tissues, as well as reduction of pancreatic β-cell function leading to impaired insulin secretion (37, 38). Moreover, the enhanced intracellular and hepatic lipid accumulation and lipolysis as a consequence of hypercortisolism could in turn contribute to the development of insulin resistance (5). Mancini et al. revealed a positive correlation between fasting glycemia and the degree of cortisol excess in CS (23). However, as previously reported (20), no association between serum or urine cortisol levels and the presence of IGM or any glycemic parameters was found in the present study, suggesting that the influence of hypercortisolism on glucose metabolism might be multifactorial and indirect. Different sample size and genetic background of the studied populations may also account for the disagreement of the relationship between GC level and occurrence of IGM.

In agreement with previous studies (23), the prevalence of baseline hypertension was significantly correlated with the duration of hypercortisolism, visceral obesity, and the TG level. An independent association was detected between baseline TG and SBP in the present study, even after adjustment for other potential confounding variables, indicating hypertriglyceridemia might be a pathogenic factor for hypertension in CD. Multiple studies have demonstrated the relationship between hypertension and dyslipidemia in the non-CD population (39, 40). In the Physicians’ Health Study, TC, non-HDL-c, and the TC/HDL-c ratios predicted the onset of hypertension in 3110 men without self-reported hypertension (41). Furthermore, a 1-SD increase in serum TG concentrations was associated with a 1.8-fold higher risk of incident hypertension in middle-aged men (42), while statin therapy leads to a considerable reduction in SBP in hyperlipidemia patients (43). In a recent study, SBP values were found positively correlated with lipids profiles, including TC, TG, LDL-c, apolipoprotein A (apoA), and apoB in pediatric patients with CS (19).

The pathophysiological mechanisms of hypertension in CS are complex and still not well-known, in which GC excess plays a vital role by activating RAS, inhibiting vasodilatation, and exerting simulated mineralocorticoid activity (4). The increased circulating cholesterol and triglycerides have been shown to contribute to accelerated atherosclerosis (12), endothelial dysfunction (44), increased aortic stiffness (42), and reduced aortic diameter and consequently lead to high SBP (45). In this study, hypertension was found significantly correlated with atherosclerosis at the time of diagnosis of CD. Therefore, dyslipidemia could contribute to the development of hypertension in patients with CD (42). Chronic hypertension puts patients at increased risk for cardiovascular morbidity. Thus, early recognition of hyperlipidemia in CD and initiation of lipid-lowering therapy, even in the absence of hypertension, has the potential to reduce the long-term cardiovascular risk.

Most studies report an improvement in hyperlipidemia with remission of CD, although a complete normalization of lipid profile is usually not achieved (14, 22). In a longitudinal study, LDL-c levels showed a significant decrease at 1 year following remission of CD, but levels remained higher than healthy controls (22). In a recent investigation on lipid profile in pediatric CD patients, most of the lipid parameters including TC, LDL-c, HDL-c, and TG-rich particles showed significant improvement at 1-year follow-up after CD is cured, expect for HDL and ApoA1 (21). In our study, a significant reduction in the incidence of hyperlipidemia and hypercholesterolemia was observed in patients in surgical remission compared to those in non-remission, indicating the effectiveness and necessity of surgical remission in improving lipid metabolism disorders in CD. The TC and LDL-c levels were significantly decreased at follow-up post-CD remission, whereas the TG level had no significant reduction and the prevalence of hypertriglyceridemia remained unchanged. These results confirmed that some lipid abnormalities might persist even after remission of CD, suggesting that life-long follow-up is necessary for better monitoring and control of dyslipidemia. The persistent lipid abnormalities might be attributed to the persistent visceral obesity and maintained disadvantageous adipokine profile even after hyperlipidemia has normalized, which further result in persistent increased cardiovascular risk and mortality in patients with CD with remission (46).

Although most of the lipid parameters changed towards a beneficial cardiovascular direction, the HDL-c level surprisingly showed a decrease after remission of CD. This aberrant change was also observed in a previous study reporting a decrease of HDL particles with a cure of CD (21). The worsening of the HDL-c level seems to be in accordance with variable alterations of HDL-c level in CS reported in previous studies (5, 23). We hypothesized that this aberrant change of the HDL-c level might involve a decrease in HDL-c synthesis and an increase in cholesterol transport from HDL to other lipoproteins after the withdraw of hypercortisolism. However, the exact mechanism needs further investigation.

The odds of hyperlipidemia at follow-up were found positively associated with higher baseline TG levels in patients with CS in remission (47). In the present study, the persistence of hyperlipidemia after surgical normalization of hypercortisolism was positively associated with baseline TC levels, regardless of other potential mediating preoperative variables. The post-surgery hormonal replacement treatments due to hypopituitarism seem to have no effect on the persistent hyperlipidemia, even though subtle hypercortisolism has been suggested associated with increased metabolic abnormalities (45). Previous studies have shown that both growth hormone deficiency (GHD) and estrogen replacement therapy due to hypogonadotropic hypogonadism (HH) can cause metabolic complications, e.g. hyperlipidemia, hypertension, and increased risk of atherosclerosis (48–50). However, no significant impact of GHD or estrogen replacement was observed on post-surgery lipid profile recovery in our cohort. These results suggest that patients with more severe preoperative lipid disturbance may exhibit poorer postoperative lipid profile recovery. Therefore, early detection and treatment of hyperlipidemia are of paramount significance in preventing its persistence after remission.

There were several limitations to our study. First, the relatively small number of subjects and the retrospective nature of the study precludes confirmation of the causal relationships between the explored parameters and weakens the statistical power of the analysis. Besides, some clinical and laboratory assays were not routinely performed during CD evaluation; therefore, several biochemical components (e.g. insulin level) were absent in the analysis. Second, since follow-up visits were at the patients’ own expense and willingness, and some of their follow-up medical records at local hospitals were hard to require, it is difficult to achieve a sufficiently long follow-up period or fixed follow-up time points while meeting sample size requirements. In this study, the follow-up period was relatively short in some patients (median: 17.3 months, range: 6.0–52.0 months) and follow-up timing was not fixed, and only 75% (78/104) of the patients enrolled at diagnosis had complete follow-up data available for post-surgery analysis. Also, some important parameters, such as blood pressure and fasting glucose, were not measured in all patients at outpatient follow-up which limited further investigation on post-surgery metabolic patterns. Third, there is no control group in this study to compare lipid abnormalities both at baseline and post-surgery. In addition, only patients who were not on lipid-lowering drugs at the time of CD diagnosis were included in this study to avoid the potential influence of the use of lipid-lowering drugs on the study of the relationship among preoperative metabolic parameters and hypercortisolism. However, in actual clinical practice, some patients may have been on lipid-lowering medication preoperatively, so their preoperative lipid metabolism and its relationship with other parameters, as well as their postoperative lipid profile recovery may differ from the results of this study.

## Conclusions

In conclusion, impaired glucose metabolism and the extent of hypercortisolism are both independent risk factors of the preoperative occurrence of hyperlipidemia in patients with CD. The pattern of lipid abnormalities may persist after surgical remission, and the persisted hyperlipidemia is associated with higher baseline TC levels. These findings suggest that the clinical management of hyperlipidemia in CD patients should focus not only on normalizing hypercortisolism by surgery but also on the early identification and control of other associated risk factors, such as impaired glucose metabolism. Furthermore, this preliminary study may provide a lead to investigate biological mechanisms underlying the common link between lipid and glucose metabolism together and their roles in the regulation of blood pressure in CD.

## Data Availability Statement

The raw data supporting the conclusions of this article will be made available by the authors, without undue reservation.

## Ethics Statement

The studies involving human participants were reviewed and approved by Institutional review board of Peking Union Medical College Hospital, Chinese Academy of Medical Sciences. Written informed consent to participate in this study was provided by the participants’ legal guardian/next of kin.

## Author Contributions

XS performed the interpretation and analysis of the clinical data, wrote the main manuscript text and prepared figure and tables. MF designed the work, and critically revised it for important intellectual content. ZZ participated in analysis of the clinical data and revision of the manuscript. LL, XB, KD, YY, HZ, and RW participated in clinical management and investigation and collected the data. All authors contributed to the article and approved the submitted version.

## Funding

This work was supported by the Natural Science Foundation of Beijing Municipality (grant no. 7182137).

## Conflict of Interest

The authors declare that the research was conducted in the absence of any commercial or financial relationships that could be construed as a potential conflict of interest.
